# Comment on Neiser et al. Assessment of Dextran Antigenicity of Intravenous Iron Preparations with Enzyme-Linked Immunosorbent Assay (ELISA). *Int. J. Mol. Sci.* 2016, *17*, 1185.

**DOI:** 10.3390/ijms18010121

**Published:** 2017-01-10

**Authors:** Claes C. Strom, Hans B. Andreasen

**Affiliations:** Pharmacosmos A/S, Roervangsvej 30, DK-4300 Holbaek, Denmark; hba@pharmacosmos.com

**Keywords:** anaphylaxis, antidextran, intravenous iron

## Abstract

All IV iron complexes carry a risk of potentially fatal allergic type hypersensitivity reactions. The mechanism(s) behind these reactions is unknown but the limited data available suggests that classic IgE mediated allergy is exceedingly rare, if ever occurring. Iron–carbohydrate molecules are complex nano-particles and trying to reduce the risk of serious hypersensitivity to antibody binding of an artificial antibody seems meaningless. A recently published analysis of safety data from randomized clinical trials confirms the method reported by Neiser to be useless to predict reaction risk. In conclusion, the study by Neiser et al. is biased, contains no new information, and has no clinical relevance. We are concerned that the association of the authors with a commercial entity has caused a conflict of interest that biases not only the results, but the entire experimental setup against competitors. (Comment on Neiser et al. *Int. J. Mol. Sci*. **2016**, *17*, 1185, doi:10.3390/ijms17071185).

## To the Editor:

It is with interest that we have read the recent article by Neiser et al. [1]. We have a number of fundamental concerns that we would like to raise.

All the authors of the publication are employees of Vifor Pharma, a Swiss-based pharmaceutical company, promoting iron sucrose and ferric carboxymaltose. Given the lack of new data and lack of clinical relevance of the experiments, our opinion is that the article is a promotional tool aimed at casting doubt on the safety profile of competing products without relevant data to support the claims.

The Vifor author group already published almost identical experiments in 2012 [2] and their speculation on isomaltoside 1000 reactivity when bound in a complex is also not new [3]. In a letter to the editor, Professors Johannes Ring and Rudi Valanta clearly outlined the lack of clinical relevance of these experiments [4]. The critique from Ring and Valanta is valid to the current article as the approach is identical and the experiments highly similar to those of the previous publication. Interestingly, in response to the critique from Ring and Valenta, the Vifor authors acknowledged that their experiments lacked clinical relevance [5].

The European Medicines Agency recently made a thorough analysis of all IV irons available in Europe and concluded that all the irons available carry a risk of serious allergic type hypersensitivity reactions, albeit very small. The mechanism(s) behind these reactions is unknown but the limited data available suggests that classic IgE mediated allergy with iron–carbohydrate complexes is exceedingly rare, if ever occurring [6]. Iron–carbohydrate molecules are complex nano-particles and trying to reduce the risk of serious hypersensitivity to antibody binding of an artificial antibody seems meaningless.

Furthermore, even disregarding the lack of relevance of focusing solely on the carbohydrate moiety, the study's exclusive focus on anti-dextran antibodies without consideration to potential reactions to other carbohydrates used in complexes is inappropriate. The stated basis for this is that (non-iron) macromolecular dextran infusion products have been shown to give rise to very rare antibody-mediated hypersensitivity reactions. However, the authors fail to consider potential mechanisms for hypersensitivity reactions associated with other types of carbohydrates, such as those used by their own company. For example, it is not mentioned that (non-iron) starch-based infusion products have been documented to give rise to antibody-mediated anaphylactic reactions [7]. This would seem reasonable for a balanced discussion as the carbohydrate in Vifor’s own product ferric carboxymaltose is branched starch derivative [8].

With the lack of knowledge around the mechanisms behind serious hypersensitivity reactions, clinical data is needed to determine the risk of individual iron–carbohydrate complexes. Since the risk of serious hypersensitivity reactions is low, large studies are required to compare risk between products.

Interestingly, a recent comparison based on pooled prospective data from randomised clinical trials with more than 5000 patients using standardised medical terms (MedDRA) to define serious and severe hypersensitivity as suggested by the US Food and Drug Administration (FDA) found a significantly lower risk of serious and severe hypersensitivity reactions with iron isomaltoside compared to ferric carboxymaltose and iron sucrose [9].

In conclusion, the study by Neiser et al. is biased, contains no new information, and has no clinical relevance.
